# Decision Support for Mitigation of Livestock Disease: Rinderpest as a Case Study

**DOI:** 10.3389/fvets.2018.00182

**Published:** 2018-09-03

**Authors:** Judith R. Mourant, Paul W. Fenimore, Carrie A. Manore, Benjamin H. McMahon

**Affiliations:** ^1^Bioscience Division, Los Alamos National Laboratory (DOE), Los Alamos, NM, United States; ^2^Theoretical Division, Los Alamos National Laboratory (DOE), Los Alamos, NM, United States

**Keywords:** rinderpest, mechanistic model, spatial model, vaccination, culling, epidemic, mitigation

## Abstract

A versatile, interactive model to predict geographically resolved epidemic progression after pathogen introduction into a population is presented. Deterministic simulations incorporating a compartmental disease model run rapidly, facilitating the analysis of mitigations such as vaccination and transmission reduction on epidemic spread and progression. We demonstrate the simulation model using rinderpest infection of cattle, a devastating livestock disease. Rinderpest has been extinguished in the wild, but it is still a threat due to stored virus in some laboratories. Comparison of simulations to historical outbreaks provides some validation of the model. Simulations of potential outbreaks demonstrate potential consequences of rinderpest virus release for a variety of possible disease parameters and mitigations. Our results indicate that a rinderpest outbreak could result in severe social and economic consequences.

## 1. Introduction

Rinderpest has been among the most devastating livestock diseases in history (1, 2). Rinderpest virus is a virulent and highly contagious pathogen that infects many cloven-hoofed livestock and wildlife species, resulting in death rates as high as 90%. Rinderpest has ravaged cattle populations in Europe, Asia, and Africa over several centuries. Use of highly effective vaccines in the context of regional and national zoosanitary control and eradication efforts led to a world-wide effort for eradication beginning in 1994. The last wild case was reported in 2001 and rinderpest was declared officially globally eradicated in 2011. It is the first animal pathogen and only the second pathogen (after smallpox virus) that has been eradicated.

While rinderpest does not exist in the wild, several countries maintain rinderpest samples and vaccines as a hedge against reintroduction (3), although this number is decreasing with 3 African countries removing their stores in 2016 (4). Current maintenance of virus and vaccine stocks is a balancing act between risk and reward. The primary purpose of the modeling presented here is to provide quantitative assessment of the consequences of virus release.

Past and potential rinderpest outbreaks are modeled as single epidemic entities amenable to control by a single vaccine, and with a reasonably well-defined presentation and progression of disease. The rinderpest virus is an RNA virus, a member of the *morbillivirus* family; Measles virus is related by divergence in the historical era (5). Several biological features support modeling rinderpest disease as a single epidemic entity. The phylogenetic diversity of the virus is not recognized to contain distinct clades. The empirical observation that vaccination using a single strain is an effective population-level control, coupled with the comparative ease of diagnosis in advanced disease, suggests low phenotypic diversity.

One of the crucial lessons that underpins our approach to modeling rinderpest in this paper is the importance of distinguishing epidemic growth due to spread in new locations vs. continued growth in areas of older infection. This observation may seem self-evident, but consideration of past analyses (e.g., Ebola in West Africa in 2014–2015), demonstrates that models which inappropriately aggregated geography can mask vital dynamics of mitigation and disease control in areas of older infection by relatively uncontrolled epidemic growth in areas of recent infection. A number of technical choices in the formulation of our decision-support oriented epidemiological model are driven by the critical importance of separating geography (see section 2.3) from temporal dependence. Making this separation in a robust way allows for far more reliable differentiation between the fundamental processes of contagion and disease progression, and the human interventions and actions that *modulate* those dynamics. In the modern world, it is these modulations that determine the outcome of epidemics. Aiding the analyses of interventions is the central challenge of epidemiological decision support, and provides the chief drivers for the design of our model in this paper.

The simulation model includes rinderpest disease progression, geographic spread, and the ability to perform historically successful mitigations such as vaccination, culling, and transmission reduction. Both a deterministic and a hybrid stochastic-deterministic version were developed. Challenges in developing compartmental, deterministic models arise from fractions of animals present in compartments–particularly at the start of disease progression in a geographical region. Similarly, fractions of animals can cause deterministic methods to have difficulty modeling extinction events (6). Spread can occur too rapidly and vaccination appears less effective because tiny fractions of individuals can start an outbreak in a new location in deterministic SIR-type models. This drawback of deterministic models was avoided by choosing a threshold for the number of exposed individuals required to start disease propagation in a geographic region. The exact value of this threshold was determined by calibration with the hybrid stochastic-deterministic model.

We first provide an overview of historical rinderpest outbreaks relevant for predicting a novel outbreak in a naïve population outlining important factors determining the scale of consequence as impacted by various mitigations. The epidemic model is then described in detail with a discussion of its accuracy and limitations. Possible outcomes resulting from reintroduction scenarios of the rinderpest virus are presented and discussed.

### 1.1. Historical perspective of rinderpest outbreaks

We guide our modeling of rinderpest outbreaks in immunologically naïve cattle populations with consideration of historical examples provided in reviews (1, 7, 8). In reviewing this history, we pay particular attention to the rate at which the geographic extent of the epidemic increases, the overall mortality rate, and the circumstances under which the epidemic was ultimately brought under control.

In 1715, Giovanni Lancisi demonstrated effective control of rinderpest outbreaks in areas of Papal Authority through zoosanitary controls (7), such as effective separation of sick and healthy animals including removal of healthy cattle from pastures where sick animals previously resided, fumigating the clothes of shepherds, burial of carcasses in deep pits, and instantly throwing milk from sick cows into a hole in the ground. The killing of animals was preferred over treatment (9). The ability to stop a small outbreak via culling and zoosanitary methods was also demonstrated by control of a 1923 outbreak in Australia through slaughter of 3,000 cattle, sheep, goats, and pigs (10).

A particularly devastating epidemic, The Great Rinderpest Pandemic of ~1887–1898, was the first in Africa (11) and is described in detail by several authors (1, 11, 12). In eastern Africa, cattle mortality rates were 98% (12) and the Massai (Masai) were devastated by the almost complete loss of their cattle (1, 11). The epidemic spread many hundreds of miles in a single year, whether inland to the Sudan from the coast, from Kenya into Tanzania, or through Zimbabwe into South Africa (1, 11). After being stopped by the Zambesi river for ~3 years, rinderpest crossed the river and spread rapidly toward South Africa at a rate of 20 mi/day (32 km/day), roughly equal to the distance an ox cart traveled in a day. Control methods of fencing, safety corridors, armed border police, and slaughter by government order (with compensation) were implemented in Transvaal and Cape Colony (South Africa). However, by late 1896 these methods were failing (12). Research into vaccine development in South Africa led to the development of a bile and serum vaccine in 1897 that had some efficacy(12).

Motivated by this pandemic, and numerous other epidemics throughout Europe, Africa, and Asia, both vaccines suitable for mass vaccination, and molecular diagnostic techniques were developed throughout much of the twentieth century (7, 13). An intensive vaccination program in China from 1950 to 1955 combined with zoosanitary measures successfully eradicated rinderpest from China (7). In other locations, however, vaccination led to tremendous reduction in rinderpest cases, followed by a later resurgence due to incomplete eradication. Joint Project 15 (JP-15) operated in the the field in Africa from 1962 to 1976 involving 22 African countries, 17 of which had active rinderpest (14, 15). This vaccination program eliminated rinderpest from large portions of Africa. However, at its end, small pockets of rinderpest existed in both east and west Africa (1, 16). By the early 1980's these pockets had expanded to cover most of sub-Saharan Africa (1) and expensive international emergency efforts were needed to bring them under control (1, 13).

The 1994 outbreak in the Shangri-La region of Pakistan (17) shows how difficult it can be to extinguish a rinderpest outbreak in the modern era without ongoing vigilance of vaccine quality and communication and education of appropriate hygienic measures to the afflicted population. Fortunately, the inaccessibility of the region, relatively low cattle population densities, and potentially the mild pathogenicity of the viral strain, kept this outbreak confined over the 18 months it took to effectively respond and extinguish this epidemic. Nevertheless, >80% mortality rates were noted in most affected regions (17).

While some of the more dramatic epidemics were due to strains of high virulence and high reproductive number (*R*_0_), both *R*_0_ and virulence vary across rinderpest strains. Endemic propagation of rinderpest leads to the dominance of milder strains of the virus with both lower virulence and *R*_0_ (18–20). Estimates of *R*_0_ over the different strains range from 1.2 to ~5 (19).

## 2. Methods

To facilitate exploration of the effects of different disease parameters and the utility of a variety of mitigations, the model has been implemented as an interactive application using the Shiny package of R. The code can be found at https://github.com/pfenimore/rinderpest.

### 2.1. Disease progression

**Figure 1:**
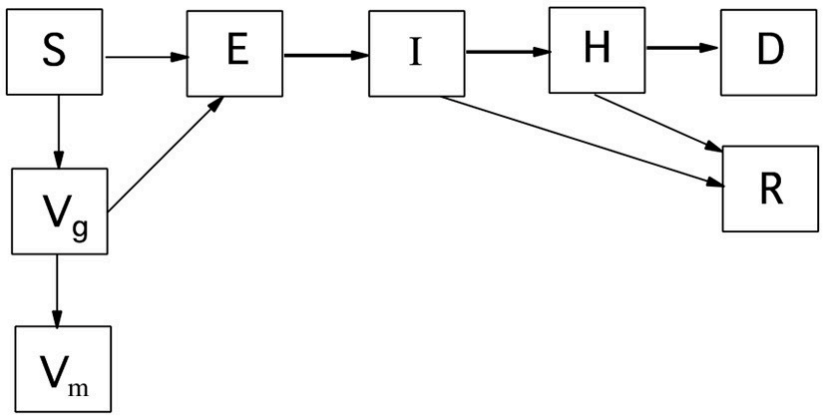
Disease progression for a single well-mixed geographical region. S, susceptible; E, exposed; I, infectious; H, seriously ill; D, dead; R, recovered; V_*g*_, vaccine given; V_*m*_, immune by vaccination. Culling is not shown, but can move cattle from states S, E, I, and H to D.

For clarity, the disease progression model is first described for a single, well-mixed geographical area without spatial spread. Figure 1 is a schematic of this disease progression with vaccination; culling is omitted. The corresponding differential equations governing the deterministic model, with culling included, are Equations 1–8. β is the transmission parameter with units of inverse time. The subscripts for disease progression rates are the starting and ending states, e.g., *k*_*IH*_ is the rate of going from the infectious and mildly ill state, I, to the seriously ill state, H. The rate susceptibles move to the vaccination-given category is *k*_*V*_. Cattle in states S, E, I, and H can be culled (not shown in Figure 1). The rate of culling from state H is *k*_*Hc*_. The total number of live cattle is N.

(1) $$\begin{array}{r} \frac{dS}{dt}=-\beta \frac{\left(I+H\right)}{N}S-k_{V}S-k_{Sc}S \end{array}$$

(2) $$\begin{array}{r} \frac{dE}{dt}=\beta \frac{\left(I+H\right)}{N}\left(S+V_{g}\right)-k_{EI}E-k_{Ec}E \end{array}$$

(3) $$\begin{array}{r} \frac{dI}{dt}=k_{EI}E-k_{IR}I-k_{IH}I-k_{Ic}I \end{array}$$

(4) $$\begin{array}{r} \frac{dH}{dt}=k_{IH}I-k_{HD}H-k_{Hc}H-k_{HR}H \end{array}$$

(5) $$\begin{array}{r} \frac{dD}{dt}=k_{HD}H+k_{Sc}S+k_{Ec}E+k_{Ic}I+k_{Hc}H \end{array}$$

(6) $$\begin{array}{r} \frac{dR}{dt}=k_{IR}I+k_{HR}H \end{array}$$

(7) $$\begin{array}{r} \frac{dV_{g}}{dt}=k_{V}S-\beta \frac{\left(I+H\right)}{N}V_{g}-k_{V_{g}V_{m}}V_{g} \end{array}$$

(8) $$\begin{array}{r} \frac{dV_{m}}{dt}=k_{V_{g}V_{m}}V_{g} \end{array}$$

### 2.2. Values of disease progression parameter

Incubation period is defined as the time between inoculation and virus secretion. Rinderpest virus can be shed a day or two before the onset of fever (21), but the level is low and shedding occurs only in a minority of animals (20). Consequently, the incubation period is taken as the time from inoculation to onset of fever. Rossiter and James give a value of 5.6 days (20). States I and H are both infectious, with I denoting the non-specific, non-severe stages of disease and H denoting severe disease. State H is defined as the infectious period after onset of characteristic mouth lesions. The animals also stop eating at this point and are obviously quite sick (14). For cattle with a virulent strain, these lesions become common roughly 3–5 days after fever onset (21).

Disease progression has varied between locales where the virus was present (19). In Table 1, the parameters used for our simulation of the 1994 Pakistan epidemic are shown along with parameters for two of the last lineages to be eradicated (19). Lineage-1 was considered to be of moderate virulence and was at one time widespread in Africa. Lineage-2 caused mild disease and was also present in Somalia. The values for time spent in states I and H in Table 1 are set to be similar to the total time spent in state I in the SEIR model of Mariner et al. (19) for lineages 1 and 2. Mouth lesions last 3 or more days (21), however, for less virulent strains a minority of animals (~20%) do not develop mouth lesions (20). Consequently, the extra time in the infectious state of the less virulent strains is assigned to state I rather than state H. The fraction of rinderpest infected cattle that die varies greatly with strain, increasing from ~2 to ~90% with virulence (20).

**Table 1:** Rinderpest disease progression parameters.

	**Pakistan**			
	**1994**	**Lineage-1**	**Lineage-2**	**References**
β (1/days)	0.31	1.1–1.4	0.17–0.19	Pakistan 1994; personal communication Paul Rossiter lineages 1 & 2; (19)
Incubation period	5.6	4.5–7	5.5–8	Pakistan 1994; (20)
1/*k*_*EI*_ (days)				lineages 1 & 2; (19)
1/*k*_*IH*_ (days)	3	1–3	4–8	estimated using (19–21)
$\frac{1}{\left(k_{HD}+k_{HR}\right)}$ (days)	3	3	3	estimated using (19–21)
Fatality fraction from H	0.9	0.36	0.06	Pakistan 1994; (17).
				Values for lineages 1 & 2 calculated using rates to R and D in Mariner's SEIR(D) model (19).

### 2.3. Geographic spread

Geographic spread is primarily modeled as being due to the movements of cattle and people directly caring for the animals. Spread to market locations can be modeled also. Only for the model of the outbreak in Pakistan is movement along roads modeled. In this section, the methods for modeling spread due to everyday activities as well as to market locations are described.

**Figure 2:**
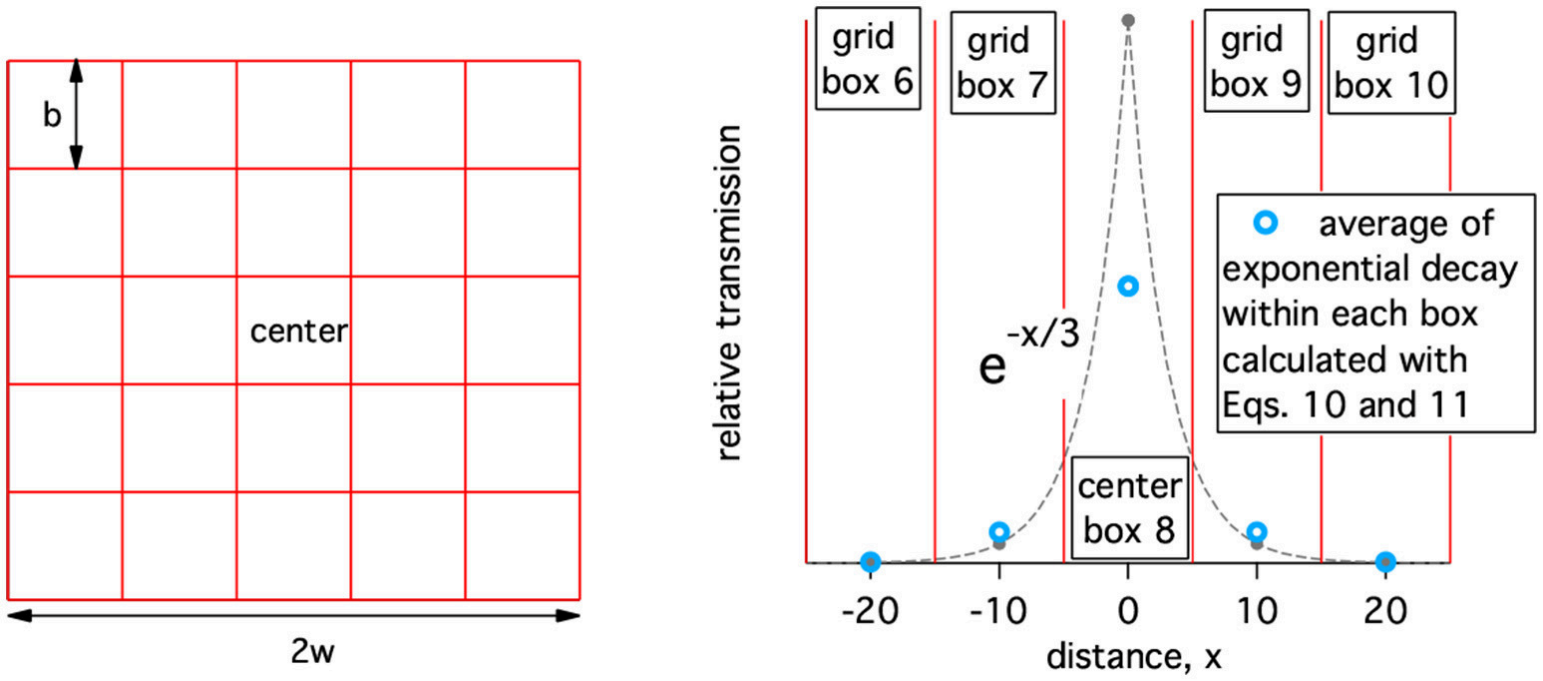
**Left:** A simple geographic grid with 25 blocks (grid elements). **Right:** Example of the relative availability of an infected animal to infected a healthy animal a distance, x, away. The width of the geographic boxes, b, is 10 and the decay of the transmission kernel is 3.

After initial incidence at a point location, disease spread is implemented on a geographic grid of cattle population with the center block containing the initial point of incidence. Geographic spread of disease is approximated as a force of infection communicating between geographical compartments. The population does not move between compartments (i.e., population grid elements). For illustrative purposes, a simple geographic grid with 25 blocks is shown on the left in Figure 2. (Note: only the grid is shown, population values are not). For the disease to spread out of the initial geographic block, some of the healthy animals in surrounding geographic blocks must be exposed to rinderpest virus. The form of the spatial spread is assumed to be similar to that of foot and mouth disease (FMD) which, as another animal disease affecting livestock, has similar spread mechanisms. The ability of FMD to spread from one location to another is known to decay rapidly with distance when movements of animals between farms and to markets is highly restricted (22, 23). We approximate this decay as exponential with distance and an example of this transmission kernel is shown on the left in Figure 2. For each block a one dimensional column in the contact-availability array is computed. In this example, a 25 by 25 matrix, *A*, is generated.

The contact-availability, *A*(*i, j*), of virus from an infectious population of animals in block *j*, to animals in block *i* is controlled by the exponentially decaying transmission kernel. Therefore, the spatial extent of the receiving block must be taken into account. This is done approximately as shown below,

(9) $$\begin{array}{r} A\left(i,j\right)\propto \int_{r\left(i,j\right)-\text{b}/2}^{r\left(i,j\right)+\text{b}/2}e^{-x/\text{d}}dx=2\text{d}e^{-r\left(i,j\right)/\text{d}}\text{sinh}\left(\frac{\text{b}}{2\text{d}}\right) \end{array}$$

where b is the size of a square block as defined in Figure 2 (left), *r*(*i, j*) is the center-to-center separation of blocks *i* and *j*, and d is the characteristic length of the exponentially decaying transmission kernel and has a value of 3 in Figure 2 (right).

For *r*(*i, i*) = 0, the contact-availability is

(10) $$\begin{array}{r} A\left(i,i\right)\propto 2\text{d}\left(1-e^{-\text{b}/\left(2\text{d}\right)}\right) \end{array}$$

The total contact availability of an animal should not depend on grid size, b. We also assume that total contact availability is independent of d and normalize it to 1. Therefore, we need,

(11) $$\begin{array}{r} \overset{n}{\underset{i=1}{\sum }}A\left(i,j\right)\leq 1 \end{array}$$

where *n* is the number of blocks in the grid. $\overset{n}{\underset{i=1}{\sum }}A\left(i,j\right)$ will be less than one if there is significant availability outside of the geographically modeled region. To facilitate normalization of the contact availability matrix, *A*(*i, j*), the simulations should be set-up such that the transmission kernel for the center box decays to approximately 0 at the edges of the modeled region. In practice, if the smallest dimension of the modeled region is 2w, w ≫ d. Therefore, the sum of all elements of the contact-availability matrix for the center block, *j* = *c*, is used to normalize the contact-availability matrix.

(12) $$\begin{array}{r} \overset{n}{\underset{i=1}{\sum }}A\left(i,c\right)=\underset{i\neq c}{\sum }2de^{-r\left(i,c\right)/\text{d}}\text{sinh}\left(\frac{\text{b}}{2\text{d}}\right)+2\text{d}\left(1-e^{-\text{b}/\left(2\text{d}\right)}\right) \end{array}$$

In addition to the non-directional spread described above, directional spread is also implemented; a location of a feed-lot, or market can be specified. The fraction of infectious spread that is directional, *f*_*D*_, is also specified by the user. If there is only one location that is a gathering point, e.g., a feedlot, then, a long distance contact availability array, *D*_*i, j*_ can be defined as,

(13) $$\begin{array}{r} D\left(i=\text{feedlot},j\right)=f_{D}D\left(i\neq \text{feedlot},j\right)=0 \end{array}$$

More generally,

(14) $$\begin{array}{r} \overset{n}{\underset{i=1}{\sum }}D\left(i,j\right)=f_{D} \end{array}$$

The total contact availability matrix is then,

(15) $$\begin{array}{r} T\left(i,j\right)=\left(1-f_{D}\right)A\left(i,j\right)+D\left(i,j\right) \end{array}$$

and

(16) $$\begin{array}{r} \overset{n}{\underset{i=1}{\sum }}T\left(i,j\right)=\left(1-f_{D}\right)\overset{n}{\underset{i=1}{\sum }}A\left(i,j\right)+\overset{n}{\underset{i=1}{\sum }}D\left(i,j\right)\\ =\left(1-f_{D}\right)\overset{n}{\underset{i=1}{\sum }}A\left(i,j\right)+\;f_{D}\leq 1 \end{array}$$

### 2.4. Mitigations

Several mitigations are implemented by time-dependent adjustment of rates in Equations 1–8; transmission control, vaccination, and culling. Mitigation implementation times are referenced to the time of disease identification. The disease is identified when the number of cases supersedes a set number specified by the user.

Transmission control reduces the interactions of virus- from infectious cattle- with healthy cattle. It is implemented as a reduction of β. This mitigation can represent, for example, keeping animals in different stalls (i.e., short range movement control) or improved hygiene by the people caring for the animals. The fraction of spread that is due to long range (directional) movement can be reduced. This could represent a ban on the transport of animals to feed-lots or markets.

Vaccination or culling is performed within user defined rings around each block containing symptomatic animals (states *I*, and *H*, but not *E*). The assumption is that once rinderpest has been detected, farmers and veterinarians will be on the lookout for animals with early symptoms. The list of geographical blocks where vaccination or culling is desired is updated once a day.

The number of vaccine doses which can be given in a day, *V*_*d*_, is a user input. In scenarios for which there is not enough vaccine to vaccinate all of the healthy population within a given radius of symptomatic cattle, an equal fraction of cattle are vaccinated in each of the chosen geographic blocks.

The rate for vaccination in a specific location is *k*_*V*_(*i*), where *i* is the index for a geographic box. In a time step, *t*_*s*_, with units of days, the number of doses available is *V*_*d*_*t*_*s*_. The total number of cattle available for vaccination, *S*_*t*_, is the sum of all susceptible cattle in the region chosen for vaccination. The number of doses administered in a geographic box, *i*, is then *V*_*d*_*t*_*s*_*S*(*i*)/*S*_*t*_. We assume that vaccination rates are much faster than the rate at which animals get sick and that culling and vaccination do not occur simultaneously in the same location. The expression for *k*_*V*_(*i*) assumes that *S*(*i*) decays exponentially with decay constant *k*_*V*_(*i*). The change in susceptibles, Δ*S*(*i*), equals the number of doses administered and is given in Equation (17).

(17) $$\begin{array}{r} V_{d}t_{s}S\left(i\right)/S_{t}=\Delta S\left(i\right)=S\left(i\right)\left(1-e^{-k_{V}\left(i\right)t_{s}}\right) \end{array}$$

Therefore,

(18) $$\begin{array}{r} k_{V}\left(i\right)=log\left(1-V_{d}t_{s}/S_{t}\right)/t_{s}. \end{array}$$

However, if the total number of doses available in a time step is greater than the number of susceptibles needing vaccination, i.e., *V*_*d*_*t*_*s*_/*S*_*t*_ > 1, this formula fails. In that case, we choose to leave an insignificant fraction of a susceptible unvaccinated. Then

(19) $$\begin{array}{r} S\left(i\right)e^{-k_{V}\left(i\right)t_{s}}=0.0001\text{ and }k_{V}\left(i\right)=-log\left(0.0001/S\left(i\right)\right)/t_{s} \end{array}$$

As with vaccination, culling can be performed in and around geographic blocks that have a symptomatic population including infectious and/or very ill animals. The user has the choice of culling all seriously ill animals (state *H*), all infectious animals (states *I* and *H*), or all animals in states *S*, *E*, *I*, and *H*. When there are not enough resources to cull all of the chosen animals, the choice of which animals to cull is done analogously to the methods for vaccination.

### 2.5. Governing equations

In the geographically-resolved simulation with transmission control, the governing equations for box (grid element) *i* are given below where *c*_*m*_ is the fractional reduction in β due to mitigation, and *r* is the fractional reduction in directional spread. We assume that cattle in state *H* are too sick to move around and hence do not infect cattle in other geographic areas.

(20) $$\begin{array}{r} \frac{dS\left(i\right)}{dt}=-k\left(i\right)S\left(i\right)-k_{V}\left(i\right)S\left(i\right)-k_{Sc}\left(i\right)S\left(i\right) \end{array}$$

(21) $$\begin{array}{r} \frac{dV_{g}\left(i\right)}{dt}=k_{V}\left(i\right)S\left(i\right)-k\left(i\right)V_{g}\left(i\right)-k_{V_{g}V_{m}}V_{g} \end{array}$$

(22) $$\begin{array}{r} \frac{dV_{m}\left(i\right)}{dt}=k_{V_{g}V_{m}}V_{g}\left(i\right) \end{array}$$

(23) $$\begin{array}{r} \frac{dE\left(i\right)}{dt}=k\left(i\right)\left(S\left(i\right)+V_{g}\left(i\right)\right)-k_{EI}\left(i\right)E\left(i\right)-k_{Ec}\left(i\right)E\left(i\right) \end{array}$$

(24) $$\begin{array}{r} \frac{dI\left(i\right)}{dt}=k_{EI}\left(i\right)E\left(i\right)-k_{IR}I\left(i\right)-k_{IH}I\left(i\right)-k_{Ic}\left(i\right)I\left(i\right) \end{array}$$

(25) $$\begin{array}{r} \frac{dH\left(i\right)}{dt}=k_{IH}I\left(i\right)-k_{HD}H\left(i\right)-k_{HR}H\left(i\right)-k_{Hc}\left(i\right)H\left(i\right) \end{array}$$

(26) $$\begin{array}{r} \frac{dD\left(i\right)}{dt}=k_{HD}H\left(i\right)+k_{Sc}\left(i\right)S\left(i\right)+k_{Ec}\left(i\right)E\left(i\right)+k_{Hc}\left(i\right)H\left(i\right) \end{array}$$

(27) $$\begin{array}{r} \frac{dR\left(i\right)}{dt}=k_{IR}I\left(i\right)+k_{HR}H\left(i\right) \end{array}$$

where,

(28) $$\begin{array}{r} k\left(i\right)=c_{m}\beta \frac{1}{N\left(i\right)}\overset{n}{\underset{j=1}{\sum }}\left[\left(1-rf_{D}\right)A\left(i,j\right)+rD\left(i,j\right)\right]I\left(j\right)+H\left(i\right) \end{array}$$

and

(29) $$\begin{array}{r} k_{EI}\left(i\right)=\left\{ \begin{array}{cc} k_{EI},\; & \text{if}E\left(i\right)>0.3/\left(26km^{2}\right)\\ 0,\; & E\left(i\right)\leq 0.3/\left(26km^{2}\right) \end{array}\right. \end{array}$$

### 2.6. Deterministic computational method

**Figure 3:**
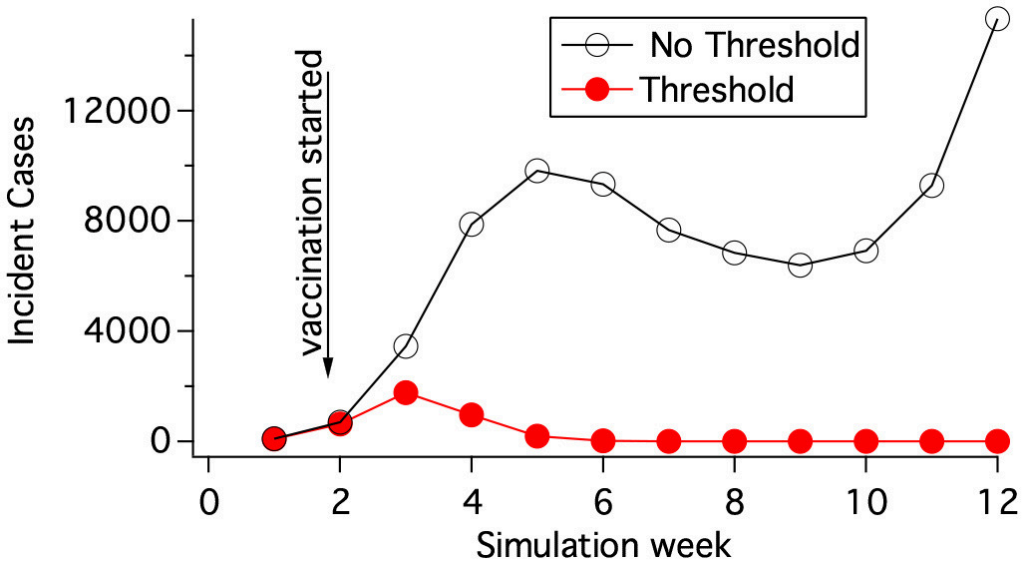
Vaccination at a rate of 7,500 vaccinations per day was begun 7 days after 5 cows had obvious symptoms (state *H*). The vaccination radius was 6 km. The population density was 60 cattle/km^2^. The threshold population in state E needed for disease progression was 0.3 cattle per grid element when calculating the red curve.

The rate coefficients, *k*_*IR*_, *k*_*IH*_, *k*_*HD*_, *k*_*HR*_ do not vary with geographic location. *k*_*EI*_(*i*), however, depends on location. If the exposed population of cattle is less than a threshold value in a grid element, *i*, then *k*_*EI*_(*i*) is set to 0 in that block and there is no disease progression. Accumulation into *E*(*i*) however can continue and may lead to disease progression in the grid element at a later time. This restriction on disease progression prevents a miniscule amount of infection from sparking a new location of incidence. Figure 3 demonstrates that the threshold population needed for disease progression in a grid element can have a large effect on whether mitigations are effective. The value of the threshold population was chosen by matching results of hybrid (section 2.7) and deterministic simulations as discussed in section 2.9.

Linearization of the contagion term in Equation 28 allows us to solve Equations 20–27 as a stepwise eigenvalue problem. We integrated the time-dependent dynamics this way because thresholding the accumulation of initial cases in a geographical cell (Equation 29) makes the net system dynamics integro-differential, obviating proofs of robustness for standard ODE solvers. The eigenvalue solution – in contrast to standard (polynomial) differential equation solvers, has a compact, well-defined inverse Laplace transform, preserving the rate-process structure of most terms in the epidemic model.

Because the *k*(*i*) are not constant during each time step, trapezoidal integration is used to improve the approximation. Approximate values of *I*, *H* and *N* at the end of the time step are first determined using *k*_*start*_. These values are then used to determine *k*_*end*_. The values for all states at the end of the time step are then calculated using *k* = (*k*_*end*_ + *k*_*start*_)/2.

### 2.7. Hybrid deterministic-stochastic computational method

Disease progression for geographic boxes in which the number of infected animals is < 50 is performed stochastically using Gillespie's τ-leap method (24). For example, to calculate the *S*→*E* transition, *k*(*i*) is calculated using Equation 28, then the number of animals transitioning from *S*→*E* is calculated by sampling a Poisson distribution with λ = *k*(*i*)*S*(*i*)*t*_*s*_, where *t*_*s*_ is the time step. The other transitions are calculated analogously.

### 2.8. Initial conditions

Initial cattle population data are from the Food and Agriculture Organization of the United Nations (25).

A single case of a disease does not always lead to an epidemic. The hybrid method captures this variability when started with a single exposed animal. The goal of this work is to determine what happens when an epidemic is started due to release of rinderpest virus. Therefore, the simulations start with a sufficient number of cases so that rinderpest is very likely to progress for several weeks. The number of starting cases is below the detection threshold that can trigger user specified mitigations. The relative number of cases in each state is based on the disease progression parameters. Specifically lineage-1 is started with 6 animals in state *E*, 2 in state *I*, and 1 in state *H*. Lineage 2 is started with 3 animals in state *E*, 2 in state *I* and 2 in state *H*. When the Pakistan 1994 parameters are used, the simulation is started with 3 animals in *E*, 2 in *I* and 1 in *H*.

### 2.9. Setting the timestep and exposed population density threshold

**Figure 4:**
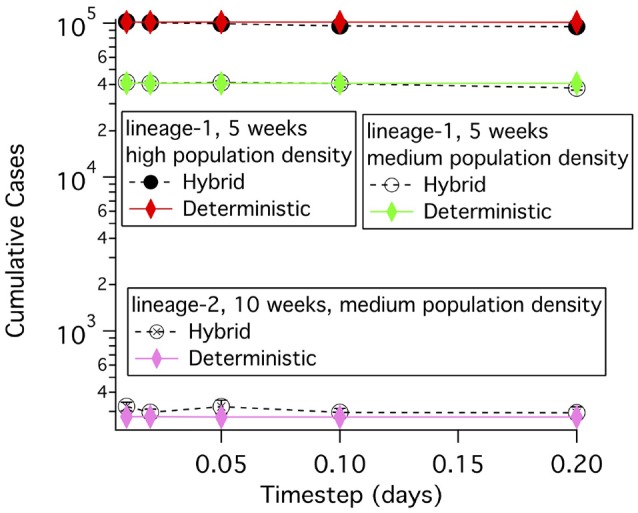
Cumulative case counts at simulation ends vs. the time step used in the simulation for both deterministic and hybrid simulations. For the hybrid model, the mean and standard deviation of 5 simulations each averaging 30 runs of the model are shown.

The timestep for the solution of the differential equations has to be set so that nearly identical answers are obtained if any shorter time step is used. Cumulative cases were simulated using different time steps for the lineage-1 and lineage-2 disease transmission and progression parameters in **Table 5** and for two population densities. The agreement between the hybrid simulations, which use a stochastic computation when the number of diseased animals in a grid element is small, and the wholly deterministic simulations is quite good over this large range of parameters as shown in Figure 4. For time steps of 0.02 and 0.05 days the deterministic results are within the errors of the hybrid simulation results. Therefore, the threshold population density needed in the exposed state of 0.3 exposed cattle per 26 km^2^ is used for all deterministic simulations. The deterministic results show very little change over a range of 0.01–0.2 day time steps, with the biggest difference in results being 0.36%. Consequently, a time step of 0.2 days is used when deterministic simulations are run. A time step of 0.05 days is used when hybrid simulations are used.

## 3. Results

### 3.1. Simulating historical outbreaks

#### 3.1.1. 1994 rinderpest outbreak in pakistan

A severe epidemic of rinderpest afflicted northern Pakistan in 1994–1995 (17). The disease was recognized during the first 6 months of the epidemic. At least 40,000 animals died and possibly as many as 50,000, approximately 7,000 of which died in the first 5 months. The first reported case was in Parri, a village south of Gilgit, in March 1994. Morbidity rates were near 100% in villages where animals were not vaccinated. The Pakistani government confirmed that the disease was rinderpest in August. Vaccination also began in August, but some of the vaccine used was later found to be subpotent and many vaccinated animals were afflicted by rinderpest. By October, rinderpest was prevalent in the upper Indus watershed including the Hunza and Gilgit valleys^1^(17). The FAO and European Union then provided nearly 4 million vaccine doses. However, due to the onset of winter and poor road conditions, only limited vaccination was performed. A final vaccination effort was started in April 1995. The outbreak ended in a village to the west, Khaplu, in November 1995 (17).

In the 1994 Pakistan outbreak, the infection moved along the roads as cattle were taken to market or to relatives. Therefore long distance movement was modeled by having the epidemic moving along the roads. The road data were taken from the OpenStreetMap project^2^. The cattle trade in this region is directional, with cattle rarely traveling south from the junction of the Gilgit with the Indus river (personal communication, Paul Rossiter). Consequently, southern movement of the epidemic along roads south of this junction was forbidden in the simulation.

For the first ~160 days of the simulation, March–August of 1994, no vaccination is performed in accordance with known facts about the epidemic. Subsequently, vaccination is performed at a rate of 200 effective cattle vaccinations per day with a vaccination ring of 30 km to simulate the use of subpotent vaccine. When more effective vaccine did arrive, winter had set in and only limited vaccination was performed. Consequently, we continue at a rate of 200 effective vaccinations per day until the simulation ends in mid-winter.

Short range, isotropic movement was modeled with an exponentially decaying function with a decay constant of 1.5 km. Movement along the roads was always in a distance range of 28–42 km. The percentage of disease spread occurring via the road network is initially 0.5%. However starting in September 1994, the amount of long distance spread is reduced by a factor of 5 under the hypothesis that if people couldn't move around very well to vaccinate cattle, the virus was not spread long distances either. Parameters are summarized in Table 2. Gridded cattle population data from the Food and Agriculture Organization of the United Nations (25) were reduced by a factor of 1.3 to account for the increase in population between 1994 and 2005 (26).

**Table 2:** Pakistan Rinderpest outbreak 1994–1995: Parameters.

	**Inital value**	**Simulation time when changed**	**New value**
β (/day)	0.31	110 days	0.25
		~July 20^*^	
d, characteristic length of transmission kernel (km)	1.3	–	1.3
long distance spread (%)	0.5	110 days	0.08
		~Jul. 20^*^	
vaccinations (/day)	0	140 days	200
		~mid August	
1/e time for immunity due to vaccination (days)	5	–	5
Fraction of infected animals that die	0.9	–	0.9

* *Assuming the epidemic started in the beginning of March*.

With so many cattle in the area dying it is expected that hygiene would be improved even before confirmation that the disease was rinderpest and the commencement of vaccination. However, reports from the time indicate that hygiene was generally poor (17). Consequently, β is reduced by 20% only 30 days before vaccination is begun.

**Figure 5:**
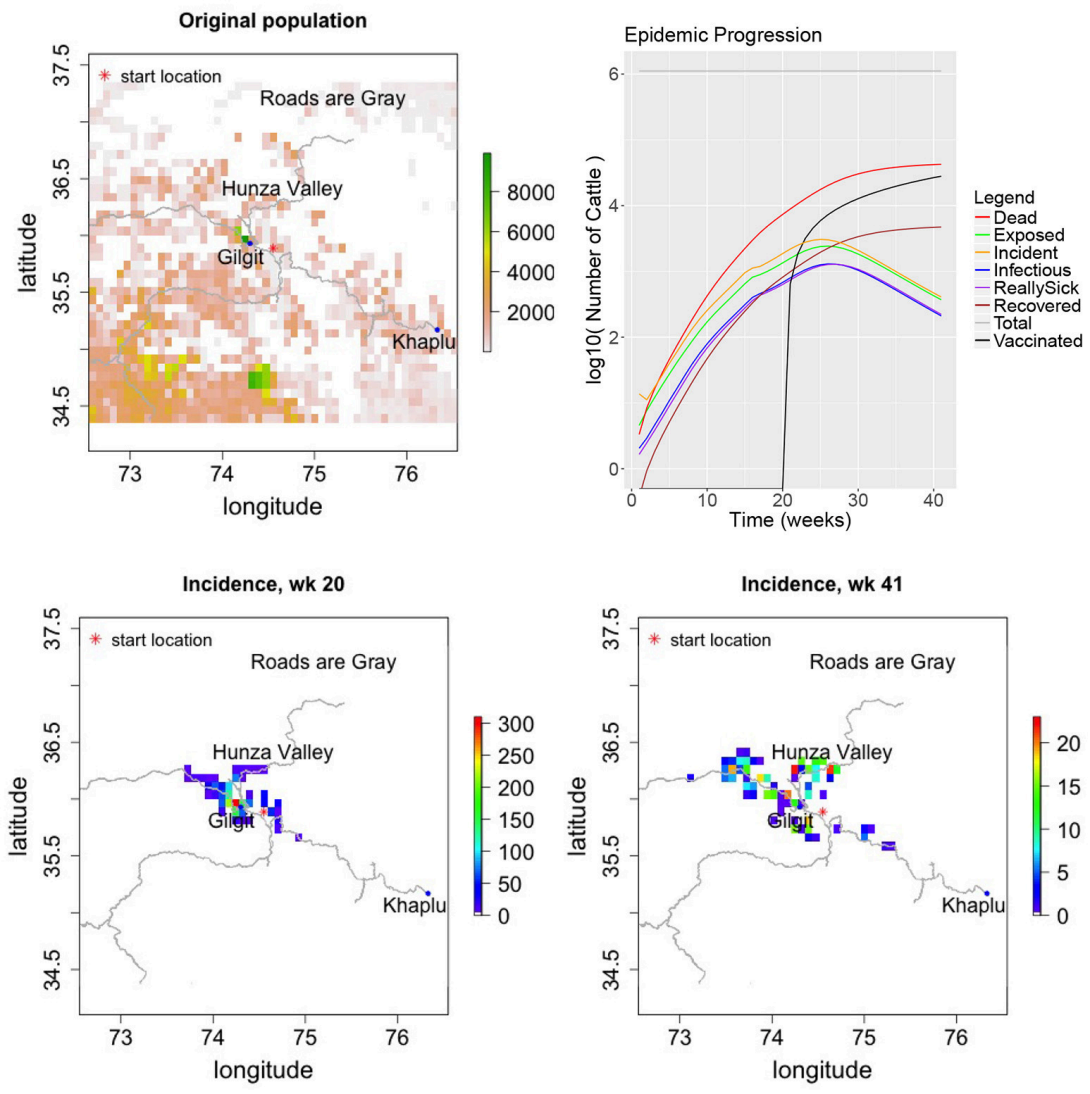
Results of simulating the 1994–1995 rinderpest outbreak in Pakistan.

The results of the simulation are consistent with known features of the epidemic. After week 20 of the simulation, i.e., in August 1994, ~7,300 cattle are dead consistent with the ~7,000 reported by Rossiter (17). Furthermore, Figure 5 shows that rinderpest was prevalent in the Hunza and Gilgit valleys as reported. At the end of the 41st simulation week, there are ~42,500 dead animals consistent with an end number of ~50,000 or less in Nov. of 1995 following a summer of effective vaccination. Therefore, geographical and temporal spread are satisfied simultaneously with reasonable parameterization.

#### 3.1.2. Fremantle, australia: control by culling and quarantine

**Figure 6:**
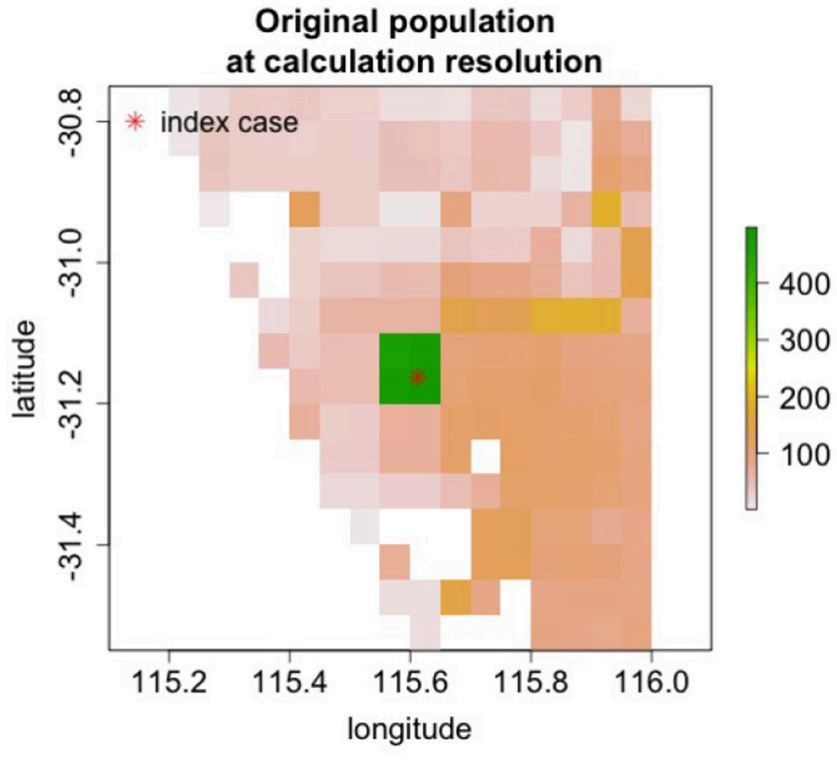
Starting population in Fremantle, Australia.

The 1923 outbreak in Fremantle, Australia near Perth was controlled by culling and quarantine (10). To roughly model this outbreak, a rinderpest epidemic starting just north of Fremantle was simulated. Since no specific information is available on the value of β, we assumed β = 1 as a stringent test of whether the epidemic could be extinguished with culling and transmission control. Mitigations began 2 days after disease detection. Culling was performed at a total rate of 1,500 animals per day with the culling radius set at 7 km and a 1 day delay between animals becoming infectious and when they are culled. Quarantine was modeled as a factor of 2 reduction in transmission. The parameters are in Table 3. The starting cattle population in Figure 6 shows that the population density is low with most of the cattle in a very localized region.

**Table 3:** Fremantle parameters.

β (1/days)	1.0
Incubation period (1/*k*_*EI*_) (days)	5.6
1/*k*_*IH*_ (days)	3
1/(*k*_*HD*_+*k*_*HR*_) (days)	3
Fatality fraction from H	0.8
Starting cases (exposed)	5
No. of animals that have had obvious	5
symptoms when rinderpest is detected	

Culling alone was able to extinguish the epidemic as shown in Table 4. With the addition of quarantine (50% transmission reduction), the epidemic was extinguished with fewer dead cattle. This level of transmission reduction alone does not end the epidemic. Without culling, the outbreak would continue and many more cattle would die after the 10 week simulation period. In the 1923 outbreak, ~3,000 animals were culled. This smaller number of culls is likely due to the lower animal population in 1923.

**Table 4:** Simulation results for Fremantle, Australia.

	**Cumulative cases**	**Dead at 10**
No mitigations	27,500 at 8 weeks^*^	14,800 at 8 weeks^*^
Cull 1,500 /day	418	14,297
Cull 1,500 /day with	144	10,820
50% transmission reduction		

* *Rounded to the nearest hundred. And still increasing*.

### 3.2. Simulation of rinderpest virus release in a naïve population

**Figure 7:**
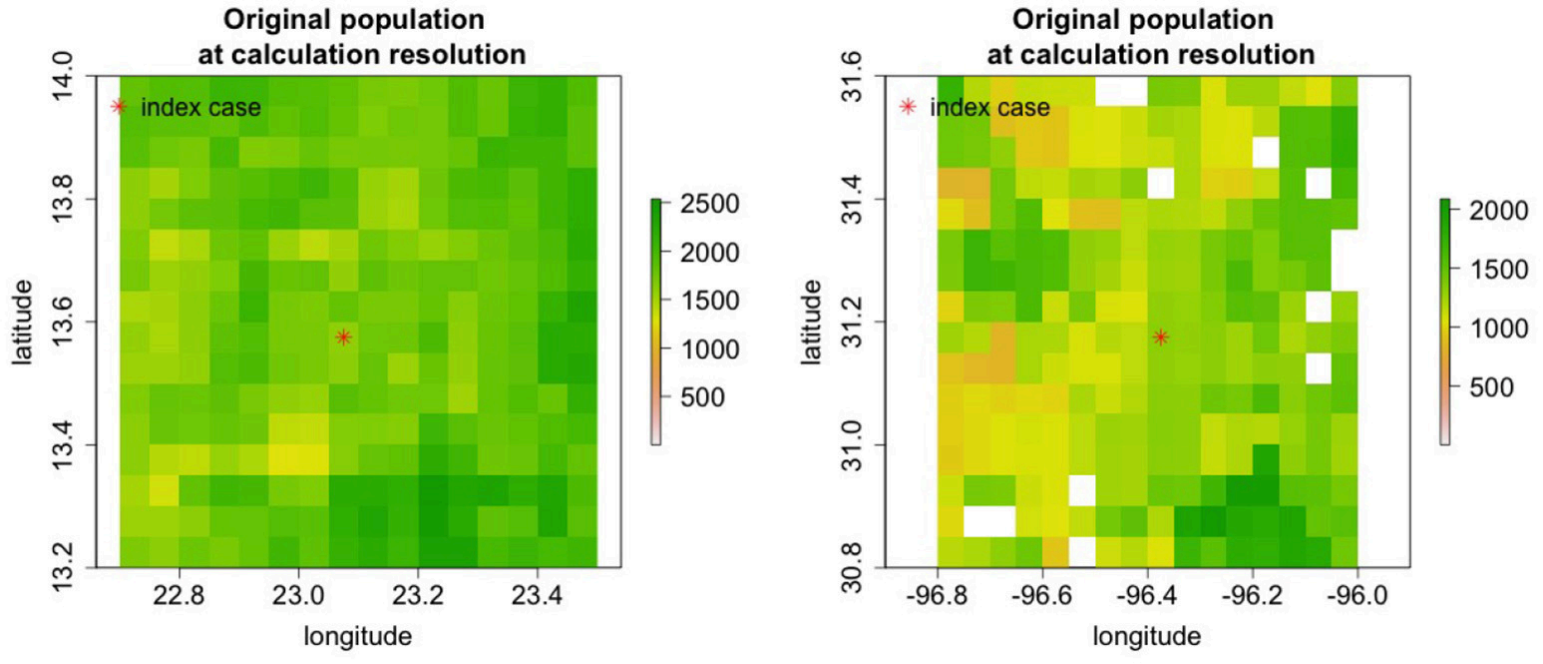
Cattle populations in two of nine regions where rinderpest introduction was simulated are shown in areas 0.8^*o*^ longitude by 0.8^*o*^ lattitide. For some simulations the area modeled was increased.

Nine regions around the world with varying population densities were modeled to understand potential epidemics resulting from the introduction of rinderpest into a naiv¨e population. The regions were chosen to have relatively uniform population densities and a few examples are shown in Figure 7.

Rinderpest varies in virulence (20), consequently simulations were performed with several parameter sets. Parameters of the strain in the 1994 Pakistan outbreak and lineage 1 and 2 from Mariner et al. (19) are shown in Table 5. For all simulations the characteristic distance for spreading was 1.3 km and the characteristic time for the vaccine to become effective was 5 days.

**Table 5:** Simulation starting parameters.

	**Lineage-1**	**Lineage-2**
β (1/days)	1.2	0.18
Incubation period (1/*k*_*EI*_) (days)	5.6	6.8
1/*k*_*IH*_ (days)	3	7
1/(*k*_*HD*_+*k*_*HR*_) (days)	3	3
Fatality fraction from H	0.36	0.06
No. of animals that have had obvious	5	5
symptoms when rinderpest is detected		

#### 3.2.1. Uncontrolled release

**Figure 8:**
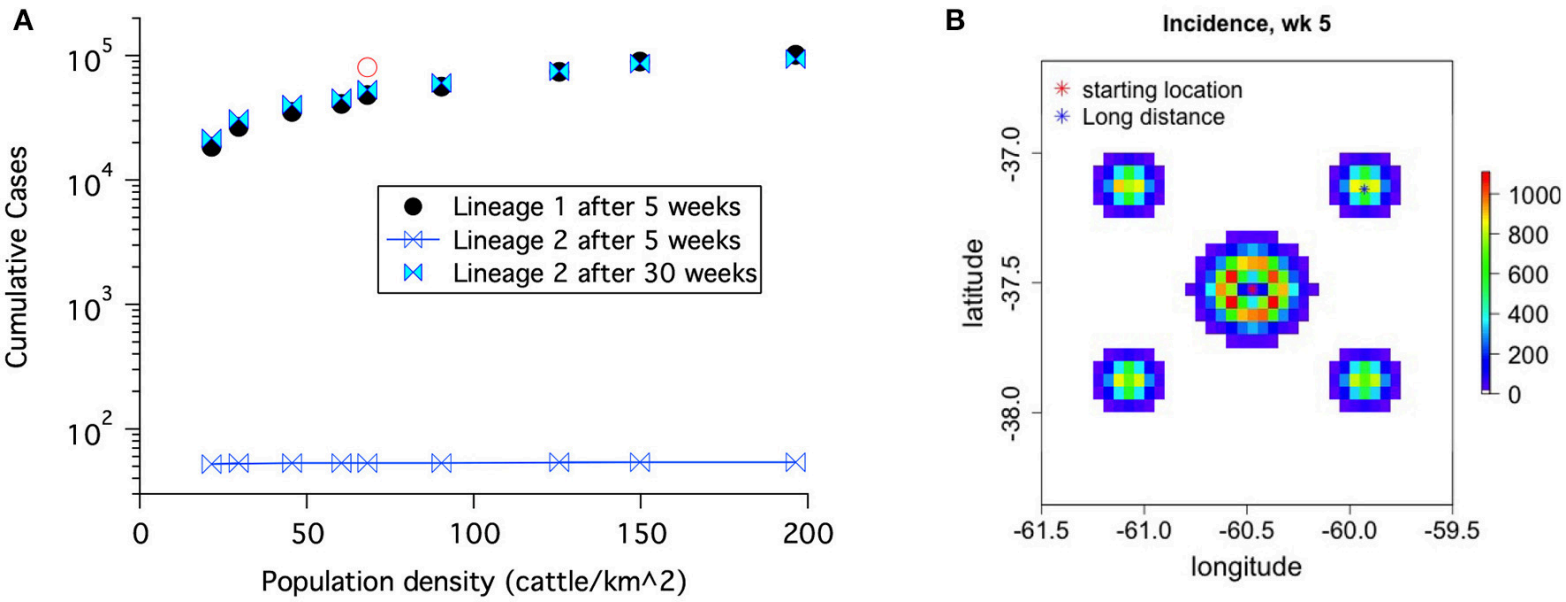
**(A)** Cumulative case counts at the end of the simulation increase with population density. **(B)** Geographical spread of disease including both local and long distance spread for lineage-1 at 5 weeks. The red open circle in **(A)** corresponds to results with the 1% spread to the four long distance locations shown in **(B)**.

Cumulative case counts at the end of simulations are shown as a function of population density in Figure 8. Simulations with lineage-1 parameters were run for 5 weeks, while simulations with lineage-2 parameters were recorded for 5 and 30 weeks. When case counts are low as for the 5 week simulations using lineage-2, the results are nearly independent of initial populations because S/N is approximately 1 (See Equations 20, 28, 23). However, when case counts rise and S/N falls in the lower population regions, a strong dependence on population density is seen; the lineage-1 results at 5 weeks and the lineage-2 results at 30 weeks in Figure 8.

Before rinderpest is detected it could spread to locations distant from the original outbreak. This type of spread occurred in the 2001 FMD outbreak (27). Once rinderpest is detected, we assume that long distance movement of cattle will be banned (and stopped). Nonetheless, the initial spread leads to a more rapidly progressing epidemic. The open circle in Figure 8 demonstrates this increase at 5 weeks for lineage-1 parameters when 1% of the spread is to long distance locations until long distance movements are stopped 2 days after rinderpest is detected.

**Figure 9:**
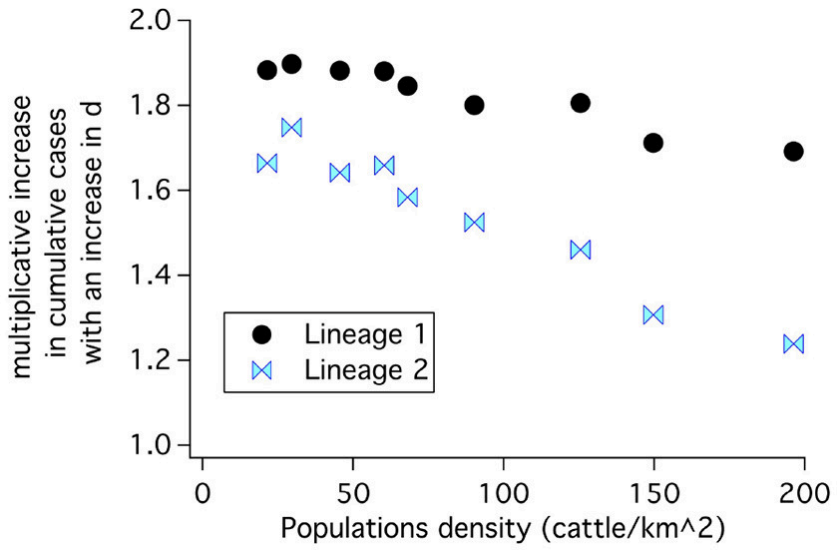
The characteristic distance of spread, d, was increased from 1.3 to 2 km. The cumulative cases at 30 weeks increased for both sets of disease parameters for uncontrolled epidemic propagation.

A final input to investigate is the characteristic distance of spread, d. Increasing d from 1.3 to 2 km increases the number of cases (after the start phase of the simulations) as shown in Figure 9. This increase is greater for areas with lower populations. This population dependence can be explained as the increased spread providing a larger susceptible population. The variation in the general trends are due to the fact that these real world populations are not homogeneous.

#### 3.2.2. Effects of mitigations

Three mitigations are modeled: reduction in transmission, vaccination, and culling. As discussed in the historical perspective section, transmission can be reduced by better hygiene and by movement control. These measures can slow an epidemic, but do not extinguish it. Vaccination and culling both have the potential to extinguish an epidemic, but the the details of implementation are critical to both the success of the effort and the final impact of the epidemic. In the following we look at how the efficacy of mitigation is affected both by controllable parameters such as the number of vaccinations per day as well as by uncontrollable parameters such as population density and characteristic spread distance. Results are presented only for lineage-1 because the results are a more stringent test of a mitigations efficacy.

##### 3.2.2.1. Vaccination

In the simulations, 10,000 doses were assumed to be available each day and the vaccination radius is set to 6 km. Vaccination started 7 days after the disease was identified which occurred after only 5 cattle showed clear signs of rinderpest. The length of time needed to extinguish the epidemic, the cumulative cases and the number of cattle immunized are given in Table 6 for several different regions with varying average cattle population densities. Ending an epidemic solely by vaccination is more difficult in higher population density regions. For the highest population densities, these vaccination conditions were not sufficient to stop the epidemic. If highly effective transmission reduction, begun only 1 day after disease identification, is combined with these vaccination conditions, then the epidemic can be extinguished as shown on the right of Table 6.

**Table 6:** Extinguishing lineage-1 epidemics by vaccination.

	**No transmission reduction**			**50% Transmission reduction**		
**Population density (cattle/km^2^)**	Vaccine doses given^*^			Vaccine doses given^*^		
		Cumulative cases^**^	**Weeks to extinguish**		Cumulative cases^**^	**Weeks to extinguish**
22	21,000	1,300	8	13,000	300	7
30	36,000	1,700	9	21,000	400	7
46	79,000	2,300	10	28,000	400	8
60	96,000	2,800	11	36,000	500	8
68	137,000	3,800	12	53,000	500	8
90	168,000	5,000	11	60,000	600	8
126	NC	NC	NC	141,000	800	10
150	NC	NC	NC	168,000	1,000	10
196	NC	NC	NC	262,000	1,600	11

* *Rounded to nearest thousand*.

** *Rounded to nearest hundred*.

*^NC^ The epidemic is not controlled within 15 weeks*.

**Figure 10:**
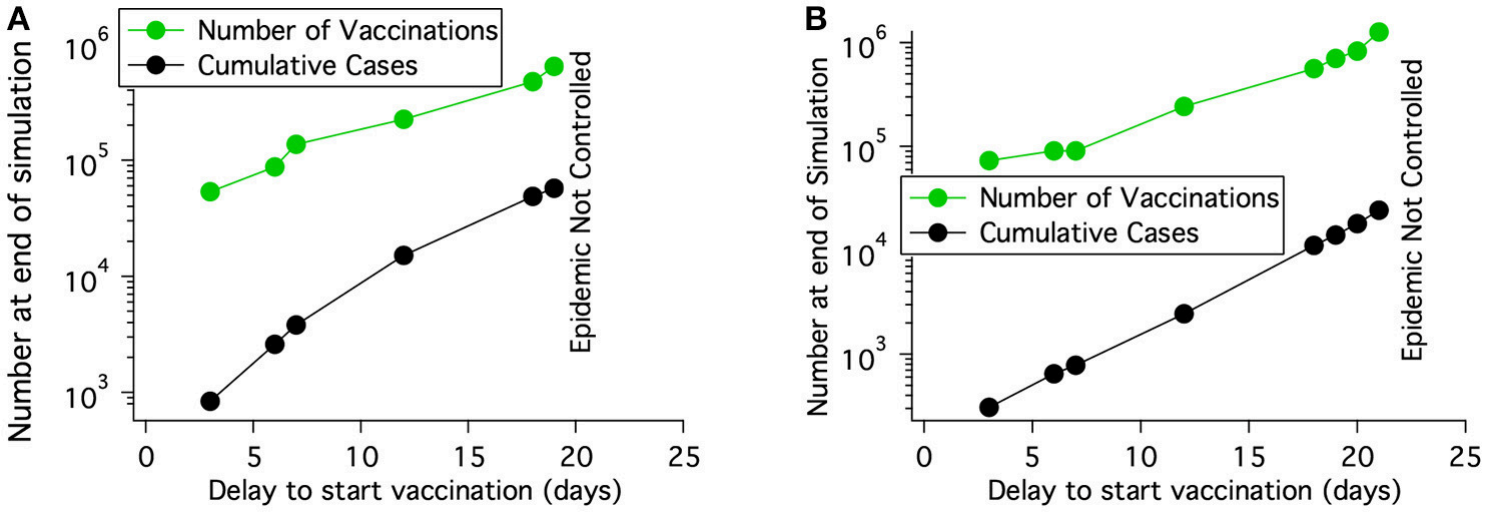
For a fairly uniform population density of 68 cattle/km^2^ and a rinderpest virus having the parameters of lineage-1, the number of vaccinations needed to control an epidemic increases rapidly with the delay in starting vaccination after rinderpest is identified. If the delay is long enough, the epidemic can not be extinguished. **(A)** No long distance spread and no transmission reduction. **(B)** Long distance spread is allowed to the locations shown in Figure 8B until 1 day after disease detection. Transmission is reduced by a factor of 2 also 1 day after disease detection.

The effect on epidemic progression of the start time for vaccination was studied. Figure 10A shows that for a region with a cattle density of roughly 68 cattle/km^2^, the number of cattle immunized goes up rapidly with the delay in starting vaccination until the available 10,000 doses/day is not enough to control the epidemic. For a population density of 126 cattle/km^2^ which was shown as not controlled in Table 6, the epidemic can be controlled if the start delay is reduced from 7 to 5 days. The length of the epidemic is reduced further as the delay is decreased. For a population density of 150 cattle/km^2^ the epidemic can only be brought under control by vaccination alone if vaccination begins after a nearly impossible delay of only 2 days after detection. Clearly, the delay in starting vaccination is a critical parameter.

All the results thus far concerning vaccination have been obtained using a characteristic spread distance of 1.3 km. While this parameter can not necessarily be controlled as part of a mitigation, it is important to know how stable our results are to the variety of geographical spread conditions which may occur in different parts of the world. Table 7 contains results for a spread distance of 2 km for the geographic area having an average population density of 68 cattle/km^2^. With this increase in geographic spread, the epidemic can no longer be controlled by the same vaccination parameters used to obtain the results in Table 6. Rather, both the number of available doses and the radius around active cases where vaccination is performed must be increased.

**Table 7:** Effects of vaccination radius and available doses for a characteristic spread distance of 2 km and average population density of 68 cattle/km^2^.

**Vaccination radius**	**Doses/day**	
	**10,000**	**13,000**
6 km	NC^*^	NC^*^
9 km	NC^*^	Controlled

* *Not controlled at 15 weeks*.

##### 3.2.2.2. Vaccination with transmission reduction

With extensive movement controls transmission of highly infectious diseases can be reduced by roughly a factor of 2 as was shown for FMD in an analysis of the 2001 outbreak (23). The right three columns of Table 6 show that it is possible to extinguish a rinderpest epidemic with vaccination under nearly ideal circumstances; (i) the disease was identified when only 5 cattle had become seriously ill and before any infected animals were transported and the disease spread to distant locations, (ii) effective vaccination was begun 7 days after disease identification and veterinarians were able to vaccinate when and where needed, (iii) hygiene and movement control reduced transmission by a factor of 2 one day after disease identification. However, if some of the circumstances are not ideal, such as the disease spreading before identification and a delay in starting vaccination then the outbreak may not be extinguished. Even in a location of medium population density (68 cattle/km^2^) more resources are needed for greater vaccination delay and with a long enough delay the epidemic can not be controlled as shown in Figure 10B.

##### 3.2.2.3. Culling

The potential to stop the lineage-1 strain of rinderpest by culling was examined for several areas with different population densities using the parameters from Table 5. The maximum rate of killing cattle was assumed to be 10,000 per day and cattle were culled regardless of disease state. The area of the geographic bins was reduced by a factor of 4 for this work, so that the radius of culls around an infectious grid element could be examined at higher resolution. In the initial examination of culling for control, the culling radius was set at 3 km, culling was started 7 days after the epidemic was detected, and the time between an animal becoming infectious and the start of culling in that grid element and the surrounding elements defined by the cull radius was set at 2 days. The left side of Table 8 shows that culling with these fairly optimal parameters does not stop the epidemic in areas with high cattle population. When very effective transmission reduction is achieved starting only 1 day after disease detection, then culling can stop the epidemic albeit with large numbers of dead cattle that were never sick.

**Table 8:** Extinguishing epidemics by culling.

	**No transmission reduction**			**50% transmission reduction**		
**Population density (cattle/km^2^)**	Dead^*^	Cumulative cases^**^	**Weeks to extinguish**	Dead^*^	Cumulative cases^**^	**Weeks to extinguish**
22	17,000	700	4	10,000	200	4
30	22,000	900	5	14,000	300	4
46	42,000	1,100	5	20,000	300	4
60	54,000	1,200	5	30,000	300	5
68	71,000	1,500	6	34,000	300	5
90	104,000	1,900	7	46,000	300	5
126	233,000	5,900	8	82,000	400	7
150	NC	NC	NC	110,000	500	7
196	NC	NC	NC	146,000	700	7

* *Rounded to nearest thousand*.

** *Rounded to nearest hundred*.

*^NC^ Not Controlled*.

**Figure 11:**
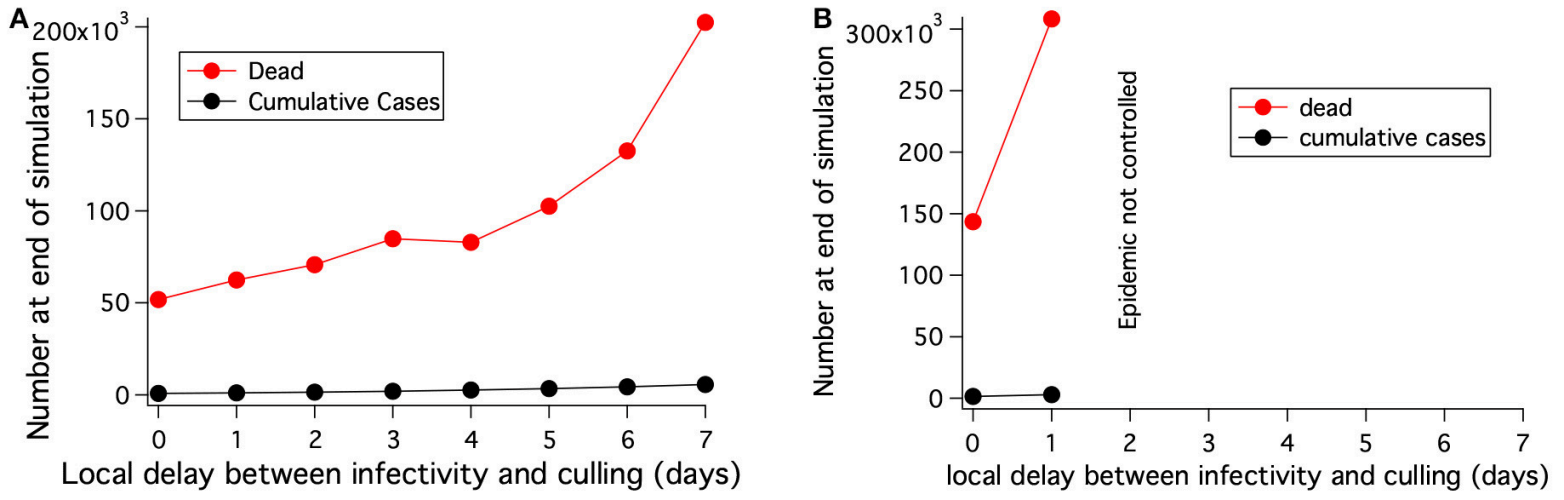
For a fairly uniform population density of 68 cattle/km^2^, the number of cattle that must be killed to extinguish an epidemic with culling depends on the delay between when the cattle at a location become infected and when they are killed. **(A)** No long distance spread and no transmission reduction. Area modeled is 0.8 × 0.8 degrees. **(B)** Long distance spread is allowed to the locations shown in Figure 8B until 1 day after disease detection. The epidemic can not be extinguished if the delay between when the cattle at a location become infected and when they are killed is 2 days or more. Area modeled is 1.3 × 2 degrees.

Figure 11 demonstrates that the delay between when the first animal in a grid element becomes infectious and when animals in that grid element and the specified surrounding area are culled is a critical parameter determining how many animals must be culled before epidemic extinction. An increase in this local delay increases the number of animals that must be culled in order to extinguished the epidemic increases. Furthermore, in the presence of initial long distance spread, Figure 11B, the epidemic can not be controlled if the delay between when the animals become infectious and when animals in that grid element are culled is 2 days or longer.

The radius of culling around locations with identified cases is a very important practical parameter. Owners of livestock generally do not want to have their apparently healthy animals slaughtered. Culling only locations where animals are sick can extinguish low transmissibility strains such as lineage-2 (results not shown), but not high transmissibility strains such as lineage-1. However, even for the high transmissibility strains, the culling radius around identified locations with infected animals does not need to be large for the relatively small characteristic distances of spread likely to hold for livestock (especially under movement restrictions). A characteristic spread distance of 2 km is used in the investigations of culling radius and an area of 1° × 1.5° was modeled. The effects of increasing the cull radius depend critically on the delay between disease identification and culling as well as on slaughter and disposal resources. Table 9 shows results for both a 7 day initial delay combined with a 3 day local delay, and a 3 day initial delay combined with a 4 day local delay. For these two scenarios different cull radii and limitations on available culling resources are considered. If an attempt is made to expand the culling area, but resources are inadequate to cull the entire area, then the number of dead animals can actually increase with “mitigation” if areas with infectious animals are not culled. If sufficient resources are available to cull the entire chosen area each day, then otherwise uncontrollable epidemics can be extinguished or reductions in the number of dead animals can be achieved. A consequence of this result is that a large cull radius is more important at the start of an epidemic when fewer resources are needed.

**Table 9:** Number of dead cattle for different culling scenarios^*^ using a fairly uniform population density of 68 cattle/km^2^.

**Initial delay to**	**Local**	**Cull**	**Culls/day**			
**start culling (days)**	**delay (days)**	**radius (km)**	**8,000**	**10,000**	**11,000**	**12,000**
7	3	3	NC	NC	311,000	245,000
7	3	5	NC	NC	278,000	239,000
7	3	7	NC	NC	517,000	295,000
3	4	3	NC	NC	NC	163,000
3	4	5	266,000	135,000	118,000	104,000
3	4	7	NC	174,000	104,000	133,000

* *The characteristic distance of spread was 2 km instead of 1.3 km as was used for most of the simulations*.

## 4. Discussion

### 4.1. Decision support

**Figure 12:**
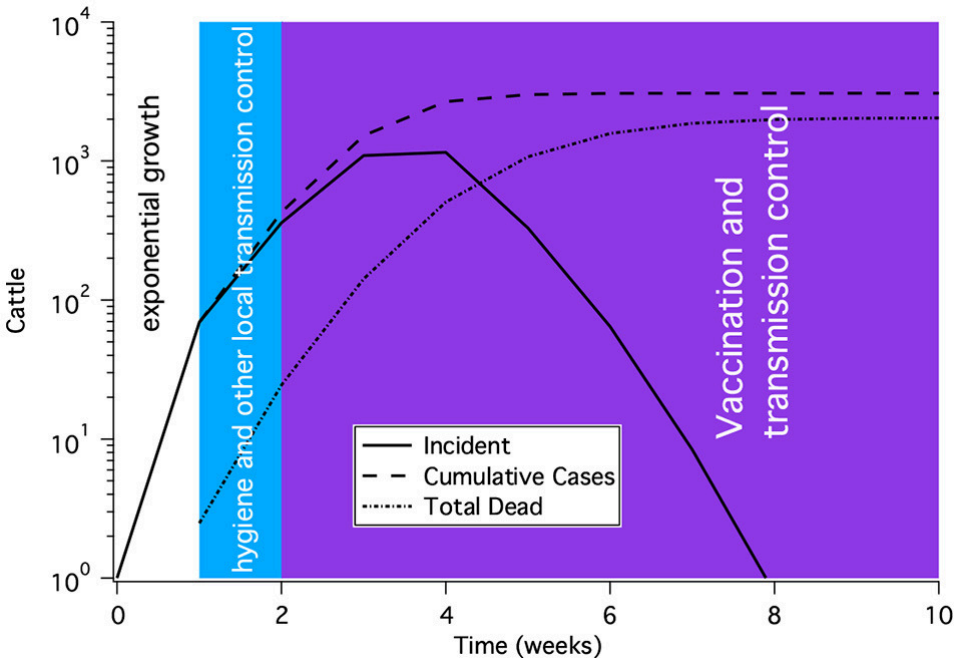
A representative time course for a rinderpest epidemic subject to modern epidemic response and control. The shape of the curve shows initial exponential rise, a slow down in growth due to transmission reduction and finally control of the epidemic after several weeks of vaccination. This time course is not meant to represent a particular outbreak, but rather to illustrate the general features of an outbreak.

Most epidemics begin with exponential growth until either there is a depletion of the susceptible population or control measures are put in place. A general scenario for incidence, cumulative cases and dead is shown in Figure 12. The first mitigation to be implemented for any disease is usually transmission reduction, often through better hygiene or separation of animals. Subsequently, either culling or vaccination is needed.

The 2001 European FMD epidemic provides insight into the practicalities of culling that are likely to arise if rinderpest were to escape from the lab. Nearly 600,000 cattle were slaughtered as a disease control measure in England (28). Based on the epidemic progression (28) and the dates when culling was performed on farms contiguous to infected farms, we estimate that the majority of culling took place over a 6 week period from about March 11th to April 29th and that on average 10,000 cattle were culled per day. Both the Netherlands and the United Kingdom experienced difficulties in safely disposing of the carcasses (28, 29). Furthermore, there was significant resistance to culling from farmers (30).

In this work concerning rinderpest, the high culling rates of the FMD epidemic were used (10,000 /day). Even with an extremely rapid start of 5 days after disease detection, culling is unable to extinguish highly contagious epidemics at the highest population densities. When culling does extinguish a weeks-to-months old epidemic, the result can be hundreds of thousands of dead cattle that were never sick.

Vaccination has the advantages of being more farmer-friendly and has no carcass disposal problems, but requires a large ready supply of effective vaccine and a means to administer it. Simulations were performed assuming 10,000 vaccinations could be performed per day. This rate is not sufficient to stop epidemics of highly virulent rinderpest in the highest cattle population areas on earth. Only in combination with highly effective hygiene control that cuts transmission in half can vaccination end epidemics in these types of scenarios. The assumption of a 50% reduction in β is based on analysis of the 2001 FMD epidemic (23). However, this large reduction may not always be achievable (17).

The presented simulations of rinderpest epidemics in naïve populations demonstrate the importance of several skills and mitigation resources. (1) The rapid detection of a rinderpest outbreak. (2) The ability to stop the movement of cattle and prevent any (further) spread to distant locations. (3) The implementation of better zoosanitary hygiene and animal isolation. If vaccination is to be used to stop an epidemic, then the ability to start an effective vaccination program of up to 10,000 animals per day on only a few days notice is needed. If culling is to end the epidemic then other resources are needed; the ability to slaughter and dispose of up to 10,000 animals per day on short (1–2 days) notice including the ability to slaughter animals on proximate sites where no animals have been diagnosed.

History demonstrates that an outbreak of a serious and highly transmissible disease such as rinderpest is likely to have severe economic and sociological consequences. As noted in section 1.1, the rinderpest outbreak in the late nineteenth century caused famine amongst the Masai people of Africa (11). Livestock have considerable social value in some parts of sub-Saharan Africa as described by Catley et al. (31). Other parts of the world are not immune from the socio-economic consequences of livestock disease. The 2001 outbreak of FMD, another serious and highly transmissable livestock disease, caused economic hardship both for farmers with herds affected by infection and/or culling as well as for otherwise unaffected farms due to movement and trade restrictions (32). The epidemic was very costly with the UK Department of Environment Food and Rural Affairs (DEFRA) spending >£3 billion (33).

### 4.2. Comparison of our techniques to other modeling methods

There is a significant body of work modeling the geographical spread of infectious disease. Much of the early work focused on spread between isolated cities and these models are sometimes referred to as metapopulation and/or patch models (34, 35, 36). However, in many areas of the world, human and animal populations are fairly contiguous and a model of well separated cites or farms is not appropriate. There is a large body of research on which transport model is best under which conditions (37, 38) and a conceptual analysis of process-driven (i.e., mechanistic) modeling frameworks has been published (39). Our rectangular tiling of population and spatial spread kernel are both simple to implement and understand. As noted in Mancy et al. (39), when working at the research–policy interface non-complex models have advantages if they can be implemented without sacrificing accuracy. Additionally, there is evidence that the exponentially decaying spread kernel is appropriate from the 2001 foot and mouth disease outbreak (23, 22).

This combination of gridded population data and a spatial transmission kernel has been previously used in modeling the spatio-temporal spread of epidemics. For example, models incorporating airline transport over the entire world have been developed (40, 41). The effects of pixel size in the grid have been investigated using a stochastic SIR model (42). A model of Rift Valley fever (43) and a model of rinderpest in the US (44) used deterministic progression and spread within “counties” combined with stochastic spread between “counties.”

One of the challenges for deterministic models is constructing realistic spatially explicit models (45). The model presented here addresses that challenge without the considerable computational cost and large data requirements of agent-based models, nor the infinite spectrum of approximate eigenvalues in a non-linear partial differential equation. Our deterministic simulation can be run in seconds for an area of 88 × 88 km on a laptop computer. Therefore, effects of a variety of mitigations can be analyzed rapidly. A screen shot of the graphical interface is shown at the end of this paper (Appendix).

## 5. Conclusions

The described simulation methods can model the time course of historical data using the known mitigation methods and disease progression parameters. Additionally, the time-dependence of the geographic extent of the simulated epidemic compared with the known extent of the historical outbreaks provides significant further constraint of the model. This geographically-resolved epi-model, developed using historical data, provides information for present-day decision-support.

Mitigation of a rinderpest outbreak without serious or even devastating effects on human society depends on disease recognition when case numbers are still small, and requires a response in a few days or 1–2 weeks depending on the transmissibility of the virus and the cattle population density. Even large-scale, effective vaccination within a week of noticing 5 cattle seriously ill with rinderpest in the worlds densest cattle population areas will not eliminate the epidemic on a timescale of weeks to months for the most virulent strains. The outbreak of a virulent strain in an area of dense but reasonably common cattle population would lead to hundreds of thousands of dead cattle assuming a nearly optimal response. Mitigation of epidemics involving less virulent strains should be possible with culling or vaccination, particularly when combined with transmission reduction. Responding to an epidemic requires resources and constant preparation including; an established and highly functional veterinary infrastructure, resources for carcass disposal and a large stockpile of effective vaccines. The perceived benefit from continued storage of rinderpest virus must be considered in the context of consequences due to a potential release of an already eradicated disease.

## Author contributions

BM and PF had the original idea for the basic structure of the epidemiological model. They also wrote the first version of the code. JM has taken the original code and made substantial revisions to both the underlying mathematical structure and to the interface based on discussions with PF, CM, and BM. CM has made critical inputs regarding the inner workings of the algorithm.

### Conflict of interest statement

The authors declare that the research was conducted in the absence of any commercial or financial relationships that could be construed as a potential conflict of interest.

## Footnotes

^1^*The World Without Rinderpest*. Available online at: http://www.fao.org/docrep/003/w3246e/W3246E06.htm (Accessed February 2016).

^2^Available online at: http://download.geofabrik.de (Accessed January 16, 2016).
